# Nickel chloride (NiCl_2_) induces endoplasmic reticulum (ER) stress by activating UPR pathways in the kidney of broiler chickens

**DOI:** 10.18632/oncotarget.7919

**Published:** 2016-03-04

**Authors:** Hongrui Guo, Hengmin Cui, Xi Peng, Jing Fang, Zhicai Zuo, Junliang Deng, Xun Wang, Bangyuan Wu, Kejie Chen, Jie Deng

**Affiliations:** ^1^ Key Laboratory of Animal Diseases and Environmental Hazards of Sichuan Province, Yaan, Sichuan, China; ^2^ College of Veterinary Medicine, Sichuan Agricultural University, Yaan, Sichuan, China

**Keywords:** NiCl_2_, ER stress, UPR, PERK, IRE1, Immunology and Microbiology Section, Immune response, Immunity

## Abstract

It has been known that overexposure to Ni can induce nephrotoxicity. However, the mechanisms of underlying Ni nephrotoxicity are still elusive, and also Ni- and Ni compound-induced ER stress has been not reported *in vivo* at present. Our aim was to use broiler chickens as animal model to test whether the ER stress was induced and UPR was activated by NiCl_2_ in the kidney using histopathology, immunohistochemistry and qRT-PCR. Two hundred and eighty one-day-old broiler chickens were divided into 4 groups and fed on a control diet and the same basal diet supplemented with 300 mg/kg, 600mg/kg and 900mg/kg of NiCl_2_ for 42 days. We found that dietary NiCl_2_ in excess of 300 mg/kg induced ER stress, which was characterized by increasing protein and mRNA expression of ER stress markers, e.g., GRP78 and GRP94. Concurrently, all the three UPR pathways were activated by dietary NiCl_2_. Firstly, the PERK pathway was activated by increasing eIF2a and ATF4 mRNA expression. Secondly, the IRE1 pathway was activated duo to increase in IRE1 and XBP1 mRNA expression. And thirdly, the increase of ATF6 mRNA expression suggested that ATF6 pathway was activated. The findings clearly demonstrate that NiCl_2_ induces the ER stress through activating PERK, IRE1 and ATF6 UPR pathways, which is proved to be a kind of molecular mechanism of Ni- or/and Ni compound-induced nephrotoxicity.

## INTRODUCTION

Ni is ubiquitously in our environment, and exists various forms of Ni compounds in soil, water, air and living organisms [1]. Ni is an ubiquitous transition metal that is industrially applied in many forms, which inevitably leads to a high degree of occupational and environmental exposure [2]. This widespread extraction and use increase Ni concentrations in biogeochemical cycles and enhance human exposure to Ni and Ni compounds through environmental contamination and occupational exposure. Ni and Ni compounds have long been recognized to cause adverse health effects including neurotoxicity, hepatotoxicity, nephrotoxicity, genetoxicity, reproductive toxicity and increase in risk of cancer [3-8].

There are several studies on oxidative stress, apoptosis and inflammation induced by Ni and Ni compounds in *vivo* and *vitro* have been reported [9-12]. In human, Ni can cause sensitivity, allergic skin reaction and cancer [13]. Oral NiCl_2_ can induce hepatic apoptosis and decrease liver weight and body weight in mice [4]. Amudha et al. [14] have suggested that NiCl_2_ (intraperitoneally) disrupts antioxidant system and induces kidney damage in rats. Our studies have also shown that dietary NiCl_2_ in 300 mg/kg and over can cause histopathological lesions, immunotoxicity, oxidative damage, apoptosis and cell cycle arrest in the kidney, thymus, spleen, small intestine, cecal tonsil and bursa of Fabricius of broiler chickens [15-31]. In the vitro studies, NiONPs induce human bronchial epithelial cell toxicity through increasing SIRT1-mediated apoptosis [32]. Pan et al. [33] have reported that NiCl_2_ induces apoptosis in human bronchial epithelial BEAS-2B cells. And Ni NPs reduce mitochondrial function and induces the leakage of LDH in dose- and time-dependent manner in human lung epithelial A549 cells [34]. NiONPs and NiSO_4_ can cause pulmonary inflammation through increasing IL-6 and IL-8 protein expression levels after 24 h treatment [35-37].

ER accurately ensures proteins to be folded and assembled before proteins are sent to other organelles [38]. Environmental and genetic factors that disrupt ER function cause an accumulation of misfolded and unfolded proteins in the ER lumen, which is termed ER stress [38]. ER stress leads to UPR which is a major hallmark of cytotoxicity [39]. To date, three UPR pathways have been documented: PERK, IRE1, and ATF6 [40]. Both PERK and IRE1 contain cytoplasmic kinase domains, which are well known to be activated by homodimerization and autophosphorylation in the presence of ER stressors [41]. In the case of ATF6, accumulation of unfolded proteins induces ATF6 transition from ER to the golgi, where it is cleaved by two transmembrane proteins, e.g., Site1 and Site2 proteases [42]. Cleaved ATF6 produces a cytoplasmic protein that acts as an active transcription factor. Short-term ER stress events lead to pro-survival transcriptional activities through UPR pathways. When cells undergo irreversible ER stress, UPR pathway eliminates damaged cells by apoptosis [39, 43, 44]. At present, it has been reported that As, Cd, Ag, Mn and Cu can induce ER stress [45-51]. However, there are no studies on Ni and Ni compounds-induced ER stress, except a report that nickel acetate can induce ER stress and increase CHOP protein expression in the NRK52E and the Hepa-1c1c7 [52]. Also, there are no reports on molecular mechanism of Ni and Ni compounds-induced ER stress in animals and human beings.

The objective of this study was to determine potential mechanisms of NiCl_2_-induced ER stress in kidneys of broiler chickens. We monitored the mRNA and protein expression of GRP78 and GRP94 which were the markers of ER stress. We also measured the mRNA expression of UPR pathways, e.g., PERK, IRE1 and ATF6 pathways.

## RESULTS

### Histopathological changes

**Figure 1:**
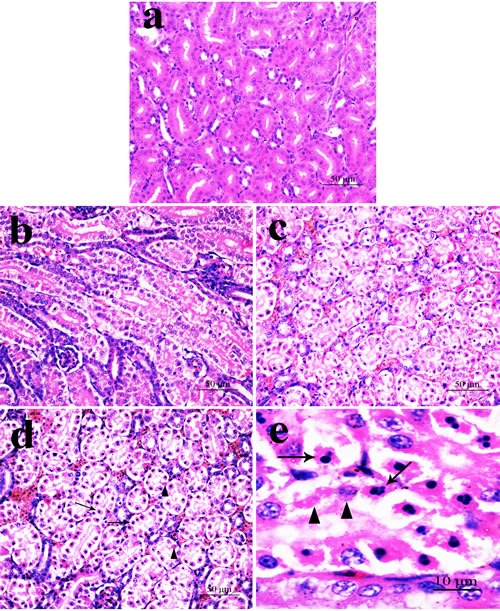
Histopathological changes in the kidney at 42 days of age (HE) **a.** Control group. No changes are observed; **b.** 300 mg/kg group. Tubular cells show granular and vacuolar degeneration. Few necrotic tubular cells and apoptotic tubular cells are also observed. **c.** 600 mg/kg group. Tubular cells show marked granular and vacuolar degeneration. Also, some necrotic tubular cells and apoptotic tubular cells are observed. **d.** 900 mg/kg group. A large number of necrotic tubular cells (▲) and apoptotic tubular cells (↑) are observed. e. Necrotic tubular cells (▲) and apoptotic tubular cells (↑) are observed.

The results were shown in the reference [25] and in Figure 1.

NiCl_2_ resulted in does- and time-dependent histopathological changes in the kidney, including tubular granular degeneration, vacuolar degeneration, necrosis and apoptosis. In the granular and vacuolar degenerated tubular cells, tiny particles and small or large vacuoles were appeared in the cytoplasm. Karyorrhexis, karyolysis and hypochromatosis were appeared in the necrotic cells. In the apoptotic cells, cytoplasm was intensely eosinophilic, and nuclei were shrunken, dense, ring-shaped and crescentic. Apoptotic bodies were also observed.

### Effect of NiCl_2_ on ER stress markers in the kidney

We first examined whether NiCl_2_ could induce ER stress in the kidney. We examined the mRNA and protein expression of GRP78 and GRP94 which were the markers of ER stress.

**Figure 2:**
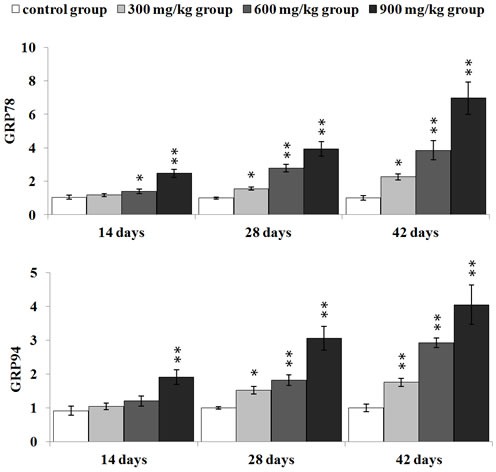
Changes of GRP78 and GRP94 mRNA expression levels at 14, 28 and 42 days Data are presented with the mean ± standard deviation (***n*** = 5) *P < 0.05, compared with the control group; ** P < 0.01, compared with the control group.

As shown in Figure 2, GRP78 mRNA expression was significantly higher (*p* < 0.05 or *p* < 0.01) in the three NiCl_2_-treated groups from 28 to 42 days of age, and in the 600 and 900 mg/kg group at 14 days of age than that in the control group. GRP94 mRNA expression was significantly increased (*p* < 0.05 or *p* < 0.01) in the three NiCl_2_-treated groups from 28 to 42 days of age and in the 900 mg/kg group at 14 days of age when compared with that in the control group.

**Figure 3:**
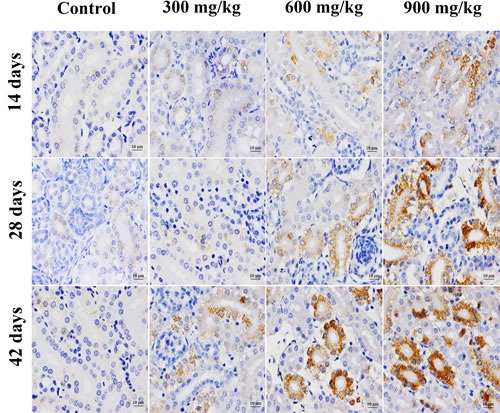
Representative images of GRP78 protein expression by immunohistochemistry at 14, 28 and 42 days of age in the kidney

**Figure 4:**
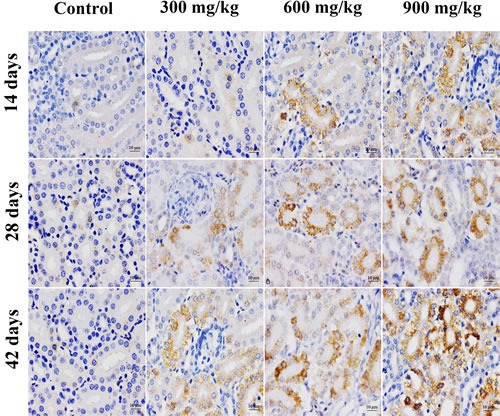
Representative images of GRP94 protein expression by immunohistochemistry at 14, 28 and 42 days of age in the kidney

**Figure 5:**
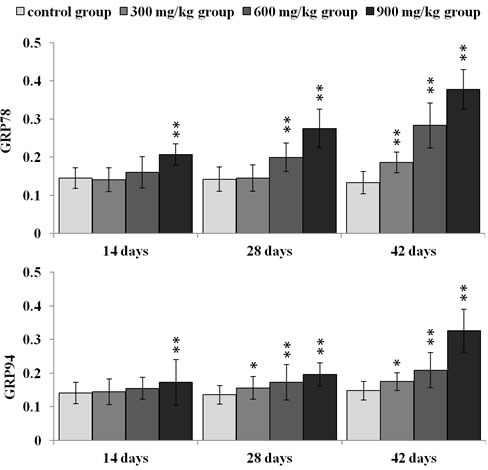
Changes of GRP78 and GRP94 protein expression levels at 14, 28 and 42 days Data are presented with the mean ± standard deviation (***n*** = 5 × 5) *P < 0.05, compared with the control group; **P < 0.01, compared with the control group.

In Figures 3, 4, 5, GRP78 protein expression was significantly higher (*p* < 0.05 or *p* < 0.01) in the 900 mg/kg group at 14 days of age, in the 600 and 900 mg/kg group at 28 days of age and in the three NiCl_2_-treated groups at 42 days of age than that in the control group. GRP94 protein expression was significantly increased (*p* < 0.05 or *p* < 0.01) in the three NiCl_2_-treated groups from 28 to 42 days of age and in the 900 mg/kg group at 14 days of age in comparison with that in the control group.

### Effect of NiCl_2_ on the three UPR pathways in the kidney

To further confirm that UPR pathways were involved in NiCl_2_-induced ER stress, we examined all the three UPR pathways: PERK pathway, IRE1 pathway and ATF6 pathway.

#### NiCl_2_ activated the PERK pathway

**Figure 6:**
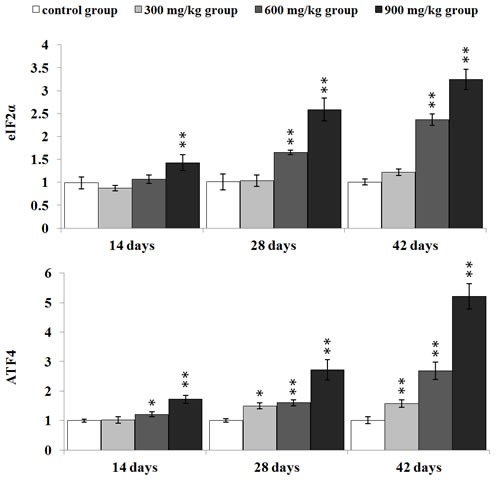
Changes of eIF2α and ATF4 mRNA expression levels at 14, 28 and 42 days Data are presented with the mean ± standard deviation (***n*** = 5) *P < 0.05, compared with the control group; **P < 0.01, compared with the control group.

In Figure 6, eIF2α mRNA expression was significantly higher (*p* < 0.05 or *p* < 0.01) in the 600 and 900 mg/kg groups from 28 to 42 days of age, and in the 900 mg/kg group at 14 days of age than that in the control group. ATF4 mRNA expression was significantly increased (*p* < 0.05 or *p* < 0.01) in the three NiCl_2_-treated groups from 28 to 42 days of age and in the 900 mg/kg group at 14 days of age when compared with that in the control group.

#### NiCl_2_ activated the IRE1 pathway

**Figure 7:**
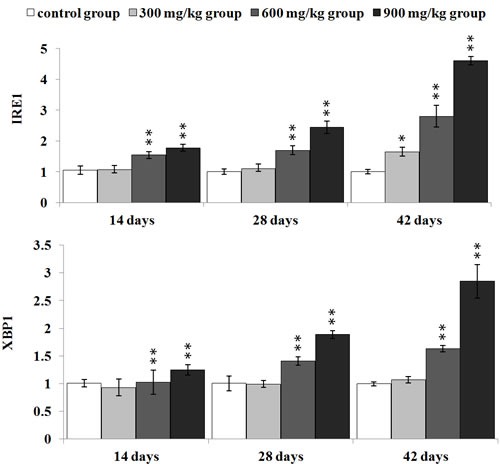
Changes of IRE1 and XBP1 mRNA expression levels at 14, 28 and 42 days Data are presented with the mean ± standard deviation (***n*** = 5) *P < 0.05, compared with the control group; **P < 0.01, compared with the control group.

IRE1 mRNA expression was significantly higher (*p* < 0.05 or *p* < 0.01) in the in the 600 and 900 mg/kg groups from 14 to 42 days of age and in the 300 mg/kg group at 42 days of age than that in the control group. XBP1 mRNA expression was significantly increased (*p* < 0.05 or *p* < 0.01) in the 600 and 900 mg/kg groups from 14 to 42 days of age in comparison with that in the control group, as shown in Figure 7.

#### NiCl_2_ activated the ATF6 pathway

**Figure 8:**
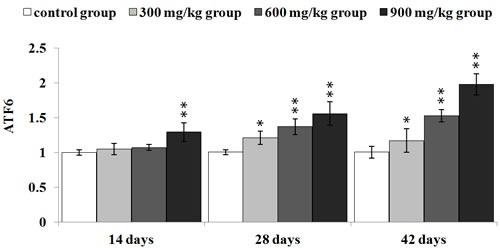
Changes of ATF6 mRNA expression levels at 14, 28 and 42 days Data are presented with the mean ± standard deviation (***n*** = 5) *P < 0.05, compared with the control group; **P < 0.01, compared with the control group.

As illustrated in Figure 8, ATF6 mRNA expression was significantly higher (*p* < 0.05 or *p* < 0.01) in the in the three NiCl_2_-treated groups from 28 to 42 days of age and in the 900 mg/kg group at 42 days of age than that in the control group.

## DISCUSSION

This study explores whether the ER stress is induced and UPR pathways are activated by NiCl_2_ in the kidney of broiler chickens. We found consistent evidence that dietary NiCl_2_ in excess of 300 mg/kg caused ER stress, which was characterized by increasing protein and mRNA expression of ER stress markers, e.g., GRP78 and GRP94. When ER stress occurs, chaperone proteins, such as GRP78 and GRP94, are recruited to support the folding of new proteins [53]. Our findings are in agreement with the results of Hiramatsu et al. [52] who report that nickel acetate can induce ER stress and increase GRP78 protein expression in the rat renal proximal tubular cell line (NRK52E) and the mouse hepatoma cell line (Hepa-1c1c7). And, the heavy metals, such as CdCl_2_ and CoCl_2_ can induce ER stress in the rat renal proximal tubular cell line (NRK52E) and the mouse hepatoma cell line (Hepa-1c1c7) [52]. Tang et al. [48] have reported that As_2_O_3_ can trigger ER stress by increasing the protein and mRNA expression of only GRP78, not GRP94.

UPR is a defense mechanism against various cellular stress which causes accumulation of unfolded proteins in the ER [40]. We also monitored the three UPR pathways: PERK pathway, IRE1 pathway and ATF6 pathway. The chaperone proteins, such as GRP78 and GRP94, are major regulators of all three pathways. Under physiological conditions, the luminal domains of PERK, IRE1 and ATF6 proteins are bound to the ER resident chaperone GRP78 and GRP94, which keeps them inactive [40]. With the accumulation of unfolded proteins, GRP78 and GRP94 release enables PERK dimerization and activation to phosphorylate eIF2a, and then the phosphorylated eIF2a induces the translation of ATF4 mRNA [54]. In the present study, the results showed that dietary NiCl_2_ increased the eIF2a and ATF4 mRNA expression, implying that PERK pathway is one of mechanisms of NiCl_2_-induced ER stress. Wang et al. [55] have suggested that CdCl_2_ exposure significantly up-regulates the phosphorylated eIF2α protein and ATF4 mRNA expression in placenta. Also, the PERK pathway is involved in Nano-ZnO-induced ER stress in mice [56]. ATF4 promotes many adaptive responses that restore ER function and maintain cell survival [57]. And ATF4 can also promote apoptosis through regulating the CHOP and Noxa [58].

In the present study, NiCl_2_ also activated IRE1 pathway, which was characterized by increasing the IREI and XBP1 mRNA expression. GRP78 and GRP94 are released from IRE1 and permitted to dimerize, which activates XBP1 kinase and RNase activities to initiate XBP1 mRNA splice, which produces a potent transcriptional activator [59]. The XBP1 mRNA expression levels are increased in Nano-ZnO-treated mice [56]. As_2_O_3_ increases the protein and mRNA expression of XBP1 in Neuro-2a cells [45]. However, it has been reported that CdCl_2_ can't activate IRE1 signaling pathway in placenta [55].

Concurrently, the increase of ATF6 mRNA expression showed that NiCl_2_ activated the ATF6 pathway. ATF6 is transported from ER to the golgi compartment, where it is cleaved to a cytosolic fragment that migrates to the nucleus to further activate the transcription of UPR-responsive genes [60]. As_2_O_3_ can also increase ATF6 mRNA expression in FHL 124 cells [61]. Xu et al. [50] have suggested that MnCl_2_ activates both PERK and IRE1 pathway, not ATF6 pathway in brain.

**Figure 9:**
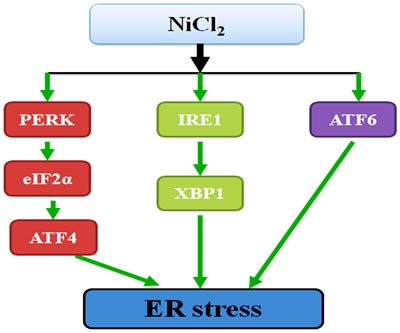
Schematic diagram of NiCl_**2**_-caused ER stress NiCl_2_ in excess of 300 mg/kg induces the ER stress. And PERK, IRE1 and ATF6 UPR pathways are all involved in NiCl_2_-induced ER stress.

Based on the results of our study and the above discussion, the mechanism of NiCl_2_-induced ER stress in the kidney is summarized in Figure 9.

In conclusion, our findings clearly demonstrate that dietary NiCl_2_ in excess of 300 mg/kg induces the ER stress through activating PERK, IRE1 and ATF6 UPR pathways, which is proved to be kind of molecular mechanism of Ni- or/and Ni compound-induced nephrotoxicity.

## MATERIALS AND METHODS

### Animals and treatment

Two hundred and eighty one-day-old healthy broilers were divided into four groups. There were seventy broilers in each group. Broilers were housed in cages with electrical heaters, and provided with water as well as under-mentioned experimental diets *ad libitum* for 42 days. The commercial broilers' growth period is about 42 days, and then they will be put into use for consumption. In this period they grow rapidly and a lot of diet will be consumed, and broilers will easily affected by diet containing metal pollutants (such as Ni). The aim of our study was to evaluate the effect of dietary NiCl_2_ on kidney in the broiler chicken of growth period.

To observe the time-dependent dynamic change, we chose three time points (14, 28 and 42 days of age) for examining histopathological injury, alterations of markers protein expression and mRNA expression levels involved in ER stress.

In this study, a corn-soybean basal diet formulated by the National Research Council [62] was the control diet. NiCl_2_ (NiCl_2_·6H_2_O, Chengdu Kelong Chemical Co., Ltd., Chengdu, China) was mixed into the corn-soybean basal diet to produce the experimental diets containing 300, 600 and 900 mg/kg NiCl_2_, respectively.

The basis of doses (300, 600 and 900 mg/kg NiCl_2_) selection: Ling and Leach reported that dietary NiCl_2_ concentrations of 300 mg/kg and over resulted in significant reduction in growth rate. Mortality and anemia were observed in chicks receiving 1100 mg/kg nickel [63]. Weber and Reid found a significant growth reduction at 700 mg/kg NiSO_4_ and nickel acetate and over [64]. Chicks fed more than 250-300 mg/kg Ni in the diet exhibited depressed growth and reduced feed intake [65]. Bersenyi et al. [66] reported that supplementation of 500 mg/kg NiCl_2_ reduced weight gain (by 10%), feed intake (by 4%) and worse FCE (by 5%) in growing broiler cockerels. According to the above-mentioned research results and our preliminary experiment, we chose the doses of 300, 600 and 900mg/kg NiCl_2_ in this study for observing the does-dependent changes.

Our experiments involving the use of broilers and all experimental procedures were approved by Animal Care and Use Committee, Sichuan Agricultural University.

### Histopathological examination

Method was appeared in the reference [25].

### Detection of protein expression in the kidney by Immunohistochemistry

Five chickens in each group were humanely sacrificed for gross examination at 14, 28 and 42 days of age. Kidneys were collected and fixed in 4% paraformaldehyde, and then processed, trimmed, and embedded in paraffin wax.

The method used in this study was described by Wu et al. in the reference [17]. Tissue slices were dewaxed in xylene, rehydrated through a graded series of ethanol solutions, washed in distilled water and PBS and endogenous peroxidase activity was blocked by incubation with 3% H_2_O_2_ in methanol for 15 min. The slices were subjected to antigen retrieval procedure by microwaving in 0.01 M sodium citrate buffer pH 6.0. Additional washing in PBS was performed before 30 min of incubation at 37°C in 10% normal goat serum (Boster, Wuhang, China). The slices were incubated overnight at 4°C with anti-GRP78 (1:200) (Cat: 3177, Cell Signaling Technology, America); anti-GRP94 (1:100) (Cat: sc-11402, Santa Cruz, America). After washing in PBS, the slices were exposed to 1% biotinylated goat anti-mouse IgG secondary antibody (Boster, Wuhang, China) for 1 h at 37°C, and then incubated with strept avidin-biotin complex (SABC; Boster, Wuhang, China) for 30 min at 37°C. To visualize the immunoreaction, slices were immersed in DAB (Boster, Wuhang, China). The slices were monitored microscopically and stopped by immersion in distilled water, as soon as brown staining was visible. Slices were lightly counterstained with hematoxylin, dehydrated in ethanol, cleared in xylene and mounted.

The expression of protein was counted using a computer-supported imaging system connected to a light microscope (OlympusAX70) with an objective magnification of 400×. The intensity of staining for each protein was quantified using Image-pro Plus 5.1 (USA). Each group was measured five slices and each slice was measured five visions and averaged.

### Detection of mRNA expression in the kidney by qRT-PCR

Kidneys from five chickens in each group were taken at 14, 28, and 42 days of age and stored in liquid nitrogen. The kidneys were homogenized in liquid nitrogen with a mortar and pestle.

According to the method described in the reference [17], total RNA was extracted from the frozen kidney powders with RNAiso Plus (9108/9109, Takara, Japan) according to the manufacturer's protocol. Next, cDNA was synthesized with a Prim-Script™ RT reagent Kit (RR047A, Takara, Japan) according to the manufacture's protocol. The cDNA product was used as a template for qRT-PCR analysis. Sequences for target genes were obtained from the NCBI database. Oligonucleotide primers were designed by use of Primer 5 software and synthesized at Takara (Dalian, China), as shown in Table 2.

**Table 1:** Abbreviations

Ni	nickel
ER	endoplasmic reticulum
UPR	unfolded protein response
NiCl_2_	nickel chloride
qRT-PCR	quantitative real-time polymerase chain reaction
GRP78	glucose-regulated protein 78
GRP94	glucose-regulated protein 94
PERK	protein kinase RNA (PKR)-like ER kinase
eIF2a	elongation initiation factor 2a
ATF4	activated transcription factor 4
IRE1	inositol-requiring enzyme 1
XBP1	X-boxbinding protein 1
ATF6	activated transcription factor 6
NiONPs	NiO nanoparticles
Ni NPs	nickel nanoparticles
LDH	lactate dehydrogenase
IL-6	interleukin-6
IL-8	interleukin-8
As	arsenic
Cd	cadmium
Ag	silver
Mn	manganese
Cu	copper
CHOP	C/EBP homologous protein
NRK52E	rat renal proximal tubular cell line
Hepa-1c1c7	mouse hepatoma cell line
CdCl_2_	cadmium chloride
CoCl_2_	cobalt chloride
As_2_O_3_	arsenic trioxide
Nano-ZnO	Zinc oxide nanoparticles
MnCl_2_	manganese chloride
FCE	feed conversion efficiency
PBS	phosphate buffer saline
DAB	3,3′-diaminobenzidine
cDNA	complementary DNA
RNAiso	RNA isolate
NCBI	national center for biotechnology information

**Table 2:** Sequence of primers used in qRT-PCR

Gene symbol	Accession number	Primer	Primer sequence(5′-3′)	Product size	Tm (°C)
GRP78	NM205491	Forward	GAATCGGCTAACACCAGAGGA	118bp	59
		Reverse	CGCATAGCTCTCCAGCTCATT		
GRP94	NM204289	Forward	CTTCGCTTCCAGTCTTCCCATC	149bp	58
		Reverse	AGAAGGCGTTCAACAAATGGTG		
Eif2a	NM001031323	Forward	GCTGCGAGTCAGTAATGGGTATAA	103bp	59
		Reverse	CTGCCAGGAAACTTGCCACA		
ATF4	AB013138	Forward	TTGATGCCCTGTTAGGTATGGAA	139BP	60
		Reverse	GGTATGAGTGGAGGTTCTTTGTTGT		
IRE1	NM001285499	Forward	TGAGGGCAATGAGAAATAAGAAGC	127bp	61
		Reverse	TGTAGGAGCAGGTGAGGGAAGC		
XBP1	NM001006192	Forward	GCGAGTCTACGGATGTGAAGGA	140bp	61
		Reverse	TGTGGAGGTTGTCAGGAATGGT		
ATF6	XM422208	Forward	GATTGTGGGCGTCACTTCTCG	142bp	57
		Reverse	TGGGATGCCAATGTTAGCCTG		
β-actin	L08165	Forward	TGCTGTGTTCCCATCTATCG	178bp	62
		Reverse	TTGGTGACAATACCGTGTTCA		

All qRT-PCR were performed by use of the SYBR^®^ Premix Ex Taq^TM^ II system (DRR820A, Takara, Japan) with on a Model C1000 Thermal Cycler (Bio Rad, USA).

Chicken β-actin expression was used as an internal reference housekeeping gene. Gene expression values from control group subsamples at 14, 28, and 42 days of age were used to calibrate gene expression in subsamples from corresponding experimental subsamples. All data output from the qRT-PCR experiments were analyzed by use of the 2^−ΔΔCT^ method [67].

### Statistical analysis

The significance of difference among the four groups of broiler chicks was assessed with variance analysis, and results were presented as mean ± standard deviation (M ± SD). The variation was measured by use of one-way analysis of variance (ANOVA) test of SPSS 16.0 for windows. *P* < 0.05 was considered statistical significance.
